# American Heart Association lipoprotein(a) discovery project: A national initiative to advance awareness and lipoprotein(a) testing for patients with cardiovascular disease

**DOI:** 10.1016/j.ajpc.2026.101664

**Published:** 2026-05-08

**Authors:** Nishant P. Shah, Zihang Gao, Shen Li, Juan Zhao, Kathie Thomas, Abha Khandelwal, Diane Osborn, Antonio B. Fernandez, Stephanie Saucier, Leslie Donato, Vlad Vasile, Nate Lebowitz, Howard Weintraub, Kurram Nasir, Kershaw Patel, Marc Bonaca, Kaavya Paruchuri, Jeremy Skinner, Kelly Gooden, Meg Yuan, Heather Gavras, Eliana Collins, Kristin Colson, David Peña

**Affiliations:** a Duke University Medical Center, Durham, NC; b American Heart Association, Dallas, TX; c Stanford University, Palo Alto, CA; d The Ohio State University, Columbus, OH; e Hartford HealthCare, Hartford, CT; f Mayo Clinic, Rochester, MN; g Hackensack Meridan Health, Fort Lee, NJ; h New York University, New York, NY; i Houston Methodist, Houston, TX; j Colorado University Anschutz, CO; k Massachusetts General Hospital, Boston, MA

Despite advancements in management, atherosclerotic cardiovascular disease (ASCVD) remains the leading cause of morbidity and mortality in the US as well as globally [1]. Residual cardiovascular risk persists even among individuals receiving optimal therapy. An important contributor to that residual risk is lipoprotein(a) (Lp[a]), an apolipoprotein B100 containing cholesterol molecule bound to apolipoprotein(a) [2]. Elevated Lp(a) shows an independent and causal effect on atherosclerosis and major adverse cardiovascular events [2]. Approximately 1 in 5 individuals exhibit elevated Lp(a) with variation of levels across sex and ethnicity. Additionally, its strong genetic basis underscores the importance of family (cascade) screening [2]. The 2026 ACC/AHA Dyslipidemia Guideline recommends that every adult have Lp(a) measured at least once in a lifetime [3].

## 1 Goals and vision of the program

The goal of the American Heart Association Lp(a) Discovery Project is to better understand health system level practice patterns for patients with elevated Lp(a) and to develop national models for Lp(a) testing that can be scaled across health systems. The primary objective is to improve the number of patients screened for Lp(a) in Get With The Guidelines®-Stroke (GWTG-Stroke) and Get With The Guidelines–Coronary Artery Disease (GWTG-CAD) registries through national professional and patient education aimed at raising awareness of the importance of Lp(a) testing. Secondarily, the project aims to understand cardiovascular risk management pathways for patients with elevated Lp(a) and to strengthen data resources by incorporating new Lp(a) data elements in GWTG.

## 2 Local challenges in implementation

Currently, <1% of individuals in the United States with measured lipid panels, including those with established ASCVD, are tested for Lp(a) [4]. Low testing rates stem from multiple factors, including limited awareness of Lp(a) as a risk enhancer, uncertainty around management strategies for elevated levels, cost and access barriers, availability of specialty clinics, competing priorities in population health, and gaps in system-level initiatives. Given the rising literature on the importance of Lp(a) as a marker of risk and several therapeutics targeting Lp(a) lowering in clinical trials, there is a critical need to raise awareness of Lp(a).

## 3 Design of the initiative

The Lp(a) Discovery Project, a three-year initiative led by the American Heart Association, recruited 10 health systems with established Lp(a) screening processes and management pathways for individuals with elevated levels. A cardiology clinical champion and stakeholders from each health system were consulted quarterly to better understand and document Lp(a) screening pathways, evaluation, and management including potential barriers. The cohort convened quarterly to share insights and discuss strategies for national dissemination of care models, including educational initiatives such as presentations at national scientific meetings, webinars, and podcasts.

To enhance data resources and improve understanding of current Lp(a) testing patterns, new Lp(a) data elements were incorporated into both GWTG-Stroke and GWTG-CAD registries. These additions aim to assess utilization of Lp(a) testing in clinical care and decision-making. Specifically, the initiative examines trends in Lp(a) testing, related treatment plans, and patient demographics to highlight gaps in cardiovascular care.

## 4 Implementation of the initiative

The American Heart Association’s GWTG-CAD and GWTG-Stroke registries are national in-hospital approaches to improving patient outcomes across cardiovascular and stroke focus areas [5] Participating hospitals collect data related to the clinical characteristics, management, and outcomes of patients admitted with stroke or transient ischemic attack (TIA) (GWTG-Stroke) or admitted due to coronary artery disease (GWTG-CAD) via the IQVIA registry platform (Parsippany, New Jersey, United States) which acts as the data collection and coordination center. Each participating hospital receives either human research approval to enroll cases without individual patient consent under the common rule, or a waiver of authorization and exemption from subsequent review by their institutional review board. Detailed information about this registry has been published elsewhere.

Data were extracted from 1/1/2023 to 12/31/2024 for GWTG–CAD and from 1/1/2023 to 5/1/2025 for GWTG–Stroke. Variation in the date ranges is due to varying data harvest timeframes. Variables analyzed included Lp(a) Value, Lp(a) Unit, Missingness of Lp(a) Value, lipid lowering therapy at discharge, and Lp(a) treatment plans. Lp(a) data elements were optional for participating hospitals upon release and became required in both registries as of October 2024. Descriptive statistics were used to characterize patients with available Lp(a) values. Continuous variables are reported as medians with interquartile ranges (IQR), and categorical variables as counts with percentages. All analyses were conducted using registry data as reported by participating sites, without imputation for missingness.

Descriptive characteristics for patients tested for Lp(a) in GWTG-CAD and GWTG-Stroke are presented in Table 1. Of the 761,748 patients in GWTG-CAD, only 2,790 (0.3%) had Lp(a) tests. Of the 9,671,350 patients in GWTG-Stroke, only 7,626 (<0.001%) had Lp(a) tests. Of the patients with an Lp(a) test, the median age in GWTG-Stroke was 67 years (IQR 21 years) and 61 years (IQR 18) in GWTG-CAD. GWTG-Stroke with Lp(a) tests comprised of 47% female while GWTG-CAD with Lp(a) tests comprised of 30% females. Additionally, of those tested for Lp(a), there were 34% non-Hispanic Black individuals in GWTG-Stroke and 20% non-Hispanic Black individuals in the GWTG-CAD. GWTG-CAD had a median Lp(a) at 79.2 (IQR 164) nmol/L and GWTG-Stroke had a median Lp(a) at 67.2 (IQR 117.6) nmol/L. Additionally, there was a high proportion of patients in GWTG-CAD with Lp(a) values ≥ 200 nmol/L (25%). The number of patients in GWTG-CAD with Lp(a) tests prescribed statins, high intensity statins, and PCSK9 inhibitors was 84.7%, 79.8%, and 1.9%, respectively at discharge. The number of patients in GWTG-Stroke with Lp(a) tests prescribed statins, high intensity statins, and PCSK9 inhibitors was 81.5%, 64.7%, and 0.3% respectively at discharge. Lp(a) treatment plan elements were released in GWTG-Stroke in February 2024 and in GWTG-CAD in April 2024. Among the patients evaluated in GWTG-Stroke, 8.5% received education on Lp(a), 0.4% were referred for lipoprotein apheresis, 5.43% were referred for lipid management, and 19.6% underwent risk factor management. In GWTG-CAD, 7.7% received education on Lp(a), 0% were referred for lipoprotein apheresis, 14% were referred for lipid management, and 17.4% underwent risk factor management.

**Table 1:** Characteristics of patients in GWTG-CAD and GWTD-Stroke with Lp(a) testing.

**Variable**	**GWTG-CAD** **Lp(a) Measured**	**GWTG-Stroke** **Lp(a) Measured**
N	2790	7626
**Age, years (median, IQR)**	61.00 (18.00)	67.00 (21.00)
**Sex**		
Female (n, %)	826 (29.61%)	3595 (47.14%)
Male (n, %)	1964 (70.39%)	4031 (52.86%)
**Race/Ethnicity (n, %)**		
Non-Hispanic White	1616 (57.92%)	3214 (42.15%)
Non-Hispanic Black	553 (19.82%)	2582 (33.86%)
Hispanic	247 (8.85%)	867 (11.37%)
Asian	214 (7.67%)	430 (5.64%)
Other/UTD	160 (5.73%)	533 (6.99%)
**Insurance (n, %)**		
Private/VA	1252 (44.87%)	2661 (34.89%)
Medicaid	365 (13.08%)	1292 (16.94%)
Medicare	792 (28.39%)	2867 (37.60%)
Self-pay/no insurance/Other/not documented/UTD	381 (13.66%)	806 (10.57%)
**Medical History (n, %)**		
Atrial Fibrillation or Atrial Flutter	144 (5.16%)	979 (12.84%)
Diabetes Mellitus	808 (28.96%)	2732 (35.82%)
Heart Failure	265 (9.50%)	810 (10.62%)
Hypertension	1790 (64.16%)	5673 (74.39%)
Prior MI	456 (16.3 %)	—
Prior CABG	152 (5.4 %)	—
Prior PCI	527 (18.9 %)	—
**Lp(a) Values (median, IQR)** nmol/L	79.20 (164.00)	67.20 (117.60)
Lp(a) Category*		
Lp(a): <75 nmol/L (n, %)	1350 (48.39%)	4132 (54.18%)
Lp(a): ≥75-124 nmol/L (n, %)	408 (14.62%)	1180 (15.47%)
Lp(a): ≥125-199 nmol/L (n, %)	348 (12.47%)	1014 (13.30%)
Lp(a): ≥200 nmol/L (n, %)	684 (24.52%)	1300 (17.05%)
**Medications at Discharge (n, %)**		
Statin at discharge	2364 (84.73%)	6217 (81.52%)
PCSK9i at discharge	52 (1.86%)	23 (0.30%)
High intensity statin†	2227 (79.82%)	4939 (64.77%)
**Lp(a) Treatment plan (n, %)**		
Patient education on Lp(a)	216 (7.74%)	648 (8.50%)
Lipoprotein apheresis	0	30 (0.39%)
Referral for lipid management	391 (14.01%)	414 (5.43%)
Risk factor management	484 (17.35%)	1494 (19.59%)

Beon C, Wang L, Manchanda V, et al. Empowering Research With the American Heart Association Get With The Guidelines Registries Through Integration of a Database and Research Tools. Circ Cardiovasc Qual Outcomes. 2024;17(9):e010967. doi:10.1161/CIRCOUTCOMES.124.010967⁵

⁎ Lp(a) values were converted from mg/dL to nmol/L WHO/IFCC SRM-2B

† High intensity statin was defined as rosuvastatin 20-40 mg and atorvastatin 40-80mg

Informed by GWTG data insights and barriers identified by the 10 participating health systems, a comprehensive educational plan was developed to support the initiative’s primary objective to improve Lp(a) screening for GWTG-Stroke and GWTG-CAD participants. The education strategy started with a webinar series that launched with a foundational session describing the mechanisms through which Lp(a) may be pathogenic, evidence for Lp(a) as an independent and causal cardiovascular risk factor, and prevalence. Subsequent webinars built on this foundation, emphasizing the clinical relevance of testing, highlighting strategies to overcome testing challenges, and disease-specific conditions such as aortic stenosis and peripheral arterial disease in relation to Lp(a). Each session was designed to progressively advance understanding, increase awareness, and identify opportunities to improve patient care through more effective testing and ASCVD management. Additionally, a podcast series was launched to explore Lp(a) through the lens of other specialties, including women’s health, neurology and primary care. These podcasts offer diverse perspectives and reinforce key messages surrounding ASCVD risk management. While the primary audience for the education series was GWTG-Stroke and GWTG-CAD participants, the education was promoted nationally and open to all audiences. Insights from this educational programming have also been disseminated at national scientific meetings, including a live panel discussion on patient empowerment and multidisciplinary collaboration, and posted to a public website (www.heart.org/lpadiscovery) further amplifying awareness and engagement.

To inform patient education resource development, 52 patients with elevated Lp(a) from diverse ethnic and socioeconomic backgrounds participated in focus groups to gain insights on lived experiences with elevated Lp(a). The focus groups revealed that patients have a lack of awareness of Lp(a) and often felt overwhelmed due to limited understanding. Many reported that their primary care clinicians were unfamiliar with Lp(a), while cardiologists and lipidologists provided more informed care. Many individuals discovered their elevated Lp(a) only after a cardiovascular event and did not know they were screened until after the result returned. Those tested in primary prevention were usually tested because they had a family member with elevated levels or were interested in understanding their level; however, requests for Lp(a) testing were often met with denial or hesitation by primary care clinicians, contributing to frustration. Once patients understood the significance of Lp(a), they became more motivated to improve their heart health.

Focus group insights informed the expansion of Lp(a) patient education resources, including infographics, discussion guides, expert videos, and patient testimonials. Lp(a) education was also added to the Heart and Stroke Helper app, a patient facing education app on optimizing heart health. Since mid-2023, patients have been able to enter their lipid values, including Lp(a), in the app.

## 5 Success of the initiative

Post-webinar surveys demonstrated substantial gains in self-reported Lp(a) knowledge and clinical application. Following the first webinar, survey respondents’ knowledge increased from minimal to 35% somewhat knowledgeable and 31% mostly knowledgeable, with 37.5% reporting adoption of the content in their practice six months later. After the second webinar, survey respondents’ knowledge rose to 44% somewhat knowledgeable and 37% mostly knowledgeable, with 63% of respondents subsequently applying what they learned to enhance Lp(a) testing. Webinar recordings have garnered more than 100,000 views and patient resources have surpassed 82,000 downloads.

## 6 Translation to other settings

To establish management pathways, consultations with health system stakeholders included process mapping to analyze testing workflows from start to finish, identifying key roles, decision points, and operational gaps. Insights gathered from these process mapping activities informed three standardized workflows tailored for cardiology, neurology, and primary care.

In cardiology, there was consensus in screening all patients for Lp(a) who were interested in knowing their individual risk. Lp(a) screening was strongly recommended for those with ASCVD, aortic stenosis, familial hypercholesterolemia, persistently elevated LDL-C, recurrent cardiovascular events, strong family history of premature ASCVD or a first degree relative with elevated Lp(a), and venous thromboembolic disease. Cardiovascular risk was correlated with the concentration of Lp(a) with low risk being <75 nmol/L (∼30mg/dL), intermediate risk being between 75-124 nmol/L (∼30-49 mg/dL), and high risk being ≥125 nmol/L (≥50 mg/dL). Management strategies for elevated Lp(a) include intensive lifestyle modification, closer monitoring and management of co-morbidities, lowering low density lipoprotein cholesterol as low as possible (at least under 70 mg/dL for primary prevention and at least under 55 mg/dL for secondary prevention), consideration of aspirin therapy, cascade screening of first degree relatives, consideration of diagnostic imaging like coronary artery calcium scoring or carotid imaging, and through shared decision-making, consideration to enroll in available clinical trials targeting Lp(a).

The screening and management strategies outlined in the cardiology workflows closely aligned with neurology and primary care. Within neurology, there was also interest in Lp(a) testing those with small vessel atherosclerosis noted in extracranial and intracranial vessel imaging. Both neurology and primary care emphasized early referral to cardiology or dyslipidemia clinics.

## 7 Summary of the experience, future directions, and challenges

The Lp(a) Discovery Project improved understanding of the importance of Lp(a) testing and adoption through national professional and patient education and enhanced Lp(a) data resources in GWTG. Educational resources and insights about Lp(a) testing practices and models across specialties will continue to be disseminated through various channels to ensure resources reach broader audiences and remain impactful well beyond the end of the project. Resources will also be shared with 20 community health centers who are implementing Lp(a) testing strategies as part of the American Heart Association Lp(a) Community Health Centers Discovery Project. The Lp(a) elements added to GWTG-Stroke and GWTG-CAD will serve as ongoing metrics to track Lp(a) testing trends and potentially different implementation strategies in both acute and post-acute care settings.

In conclusion, the Lp(a) Discovery Project developed foundational professional and patient education resources for increasing Lp(a) awareness and testing, but there remains a critical need to overcome barriers to testing and management for those with elevated levels. Strategies to increase equitable access to testing and improve education on the management of elevated Lp(a) must be implemented at the health system level with collaboration of many stakeholders, including affected individuals and front-line health care workers.

## Declaration of generative AI and AI-assisted technologies in the manuscript preparation process

During the preparation of this work the author(s) used ChatGPT in order to help create the foundation for a central figure to summarize the work. After using this tool/service, the author(s) reviewed and edited the content as needed and take(s) full responsibility for the content of the published article. No generative AI or AI assisted technology was used to write the manuscript.

## Funding source

Novartis funded the AHA Lp(a) Discovery Project.

**Figure:**
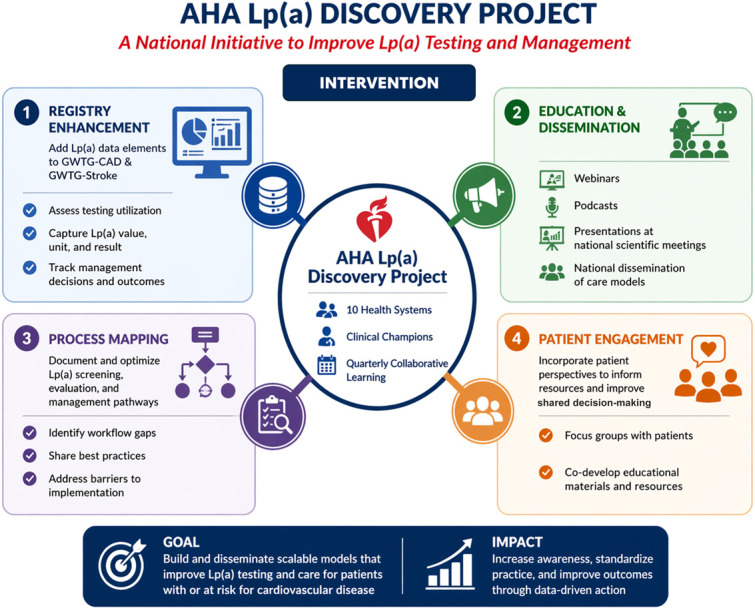
Central Figure.

## CRediT authorship contribution statement

**Nishant P. Shah:** Conceptualization, Investigation, Methodology, Project administration, Supervision, Visualization, Writing – original draft, Writing – review & editing. **Zihang Gao:** Data curation, Formal analysis, Writing – original draft, Writing – review & editing. **Shen Li:** Data curation, Formal analysis, Writing – original draft, Writing – review & editing. **Juan Zhao:** Data curation, Formal analysis, Writing – original draft, Writing – review & editing. **Kathie Thomas:** Data curation, Formal analysis, Writing – original draft, Writing – review & editing. **Abha Khandelwal:** Writing – original draft, Writing – review & editing. **Diane Osborn:** Writing – original draft, Writing – review & editing. **Antonio B. Fernandez:** Writing – original draft, Writing – review & editing. **Stephanie Saucier:** Writing – original draft, Writing – review & editing. **Leslie Donato:** Writing – original draft, Writing – review & editing. **Vlad Vasile:** Writing – original draft, Writing – review & editing. **Nate Lebowitz:** Writing – original draft, Writing – review & editing. **Howard Weintraub:** Writing – original draft, Writing – review & editing. **Kurram Nasir:** Writing – original draft, Writing – review & editing. **Kershaw Patel:** Writing – original draft, Writing – review & editing. **Marc Bonaca:** Writing – original draft, Writing – review & editing. **Kaavya Paruchuri:** Writing – original draft, Writing – review & editing. **Jeremy Skinner:** Writing – original draft, Writing – review & editing. **Kelly Gooden:** Writing – original draft, Writing – review & editing. **Meg Yuan:** Writing – original draft, Writing – review & editing. **Heather Gavras:** Writing – original draft, Writing – review & editing. **Eliana Collins:** Writing – original draft, Writing – review & editing. **Kristin Colson:** Writing – original draft, Writing – review & editing. **David Peña:** Conceptualization, Data curation, Formal analysis, Project administration, Supervision, Visualization, Writing – original draft, Writing – review & editing.

## Declaration of competing interest

The authors declare the following financial interests/personal relationships which may be considered as potential competing interests:

Nishant Shah reports financial support was provided by Regeneron Pharmaceuticals Inc. Nishant Shah reports financial support was provided by Amgen Inc. Nishant Shah reports financial support was provided by Novartis. Nishant Shah reports financial support was provided by NewAmsterdam Pharma Corporation. Nishant Shah reports financial support was provided by Eli Lilly and Company. If there are other authors, they declare that they have no known competing financial interests or personal relationships that could have appeared to influence the work reported in this paper
